# COVID-19-bedingte Online- vs. Präsenzlehre: Differentielle Entwicklungsverläufe von Beanspruchung und Selbstwirksamkeit in der Lehrkräftebildung?

**DOI:** 10.1007/s11618-022-01072-5

**Published:** 2022-03-03

**Authors:** Isabell Hußner, Rebecca Lazarides, Andrea Westphal

**Affiliations:** 1 Department Erziehungswissenschaft, Professur für Schulpädagogik, Universität Potsdam, Karl-Liebknecht-Straße 24–25, 14476 Potsdam, Deutschland; 2 Institut für Erziehungswissenschaft, Professur für Interdisziplinäre Lehr-Lernforschung und Schulentwicklung, Universität Greifswald, Steinbeckerstr. 15, 17487 Greifswald, Deutschland

**Keywords:** Selbstwirksamkeitserwartungen, Beanspruchungserleben, Lehrkräftebildung, COVID-19, Online-Lehre, Self-efficacy beliefs, Stress and burnout, Teacher training, COVID-19, Online teaching

## Abstract

Bedingt durch die COVID-19-Pandemie haben Universitäten die Lehrkräftebildung in den vergangenen Monaten rasant auf Online-Formate umstellen müssen. Für eine evidenzbasierte Weiterentwicklung der Online-Lehre sind empirische Ergebnisse zur professionellen Entwicklung von Lehramtsstudierenden erforderlich. Vor diesem Hintergrund ist es wichtig, Unterschiede zwischen Präsenz- und Onlineformaten in der Lehre zu untersuchen. In der vorliegenden Studie gehen wir daher der Frage nach, inwiefern sich Selbstwirksamkeitserwartungen und Beanspruchungserleben von Lehramtsstudierenden im Verlauf eines COVID-19 bedingten Online-Semesters sowie im Verlauf von schulpraxisbezogenen Präsenzsemestern differentiell entwickeln. An der längsschnittlichen Fragebogenstudie mit quasi-experimentellem Design nahmen *N* = 240 Lehramtsstudierende teil (*n* = 127 Online-Semester; *n* = 113 Präsenzsemester). Die Ergebnisse verweisen auf einen stärkeren Anstieg der Selbstwirksamkeitserwartungen in Präsenzformaten als im Onlineformat. In Bezug auf das Beanspruchungserleben zeigten sich keine Gruppenunterschiede. Implikationen dieser Befunde für die Weiterentwicklung der Lehrkräftebildung werden weiterführend diskutiert.

## Abstract (en)

In recent months, as a result of the COVID-19 pandemic, universities have had to rapidly move teacher training programs onto online platforms. In order to be able to develop these online formats in an evidence-based way, it is vital to have access to empirical data on the development of professional competencies in student teachers. Key to this is understanding the differences between classroom and online formats in teaching. In the present study, we therefore investigate the extent to which self-efficacy expectations and experiences of stress (burnout) in student teachers develop differently in practice-based face-to-face semesters in schools as compared to the equivalent online semester under the COVID-19 pandemic restrictions. *N* = 240 student teachers (*n* = 127 online semester; *n* = 113 face-to-face semesters) took part in the longitudinal questionnaire study with a quasi-experimental design. The results indicate a higher increase in self-efficacy expectations in face-to-face formats than in the online formats. There were no group differences with regard to experiences of stress. We discuss the implications of these findings for the further development in teacher training.

## Einleitung

Hohe Selbstwirksamkeitserwartungen sind eine bedeutsame Ressource in der Lehrkräftebildung – sie wirken einem erhöhten Beanspruchungserleben entgegen (Fives et al. 2007) und stehen mit dem pädagogischen Professionswissen im Studium in korrelativem Zusammenhang (Schulte et al. 2008). Erfolgreiche eigene Praxiserfahrungen gelten als besonders bedeutsam für die Entwicklung von Selbstwirksamkeitserwartungen in der Lehrkräftebildung (Bandura 1997). Praxisphasen werden dabei sowohl als Bereicherung (Makrinus 2012) als auch als Belastung wahrgenommen (Holtz 2014). Wie sich Selbstwirksamkeitserwartungen und Beanspruchungserleben von Studierenden entwickeln, wenn Praxiserfahrungen in der Lehrkräftebildung fehlen, ist insbesondere im Kontext der COVID-19-bedingten Online-Lehre an Universitäten eine bedeutsame Frage. Zahlreiche Studien befassen sich aktuell mit den Auswirkungen des COVID-19-bedingten Fernunterrichts auf Schülerinnen und Schüler (Huber et al. 2020; Steinmayr et al. 2021) – allerdings existieren bisher wenige Studien, die sich mit der Bedeutung der COVID-19-bedingten Online-Lehre im Hochschulbereich befassen (z. B. Osterberg et al. 2020; Schmölz et al. 2020; Scull et al. 2020). Die vorliegende Untersuchung geht vor diesem Hintergrund der Frage nach, wie sich Selbstwirksamkeitserwartungen und Beanspruchungserleben in der Lehrkräftebildung im Semesterverlauf verändern und welche Unterschiede dabei zwischen der Online-Lehre während der COVID-19-Pandemie und der Präsenzlehre mit schulpraktischem Anteil bestehen.

## Theoretischer Hintergrund

### Selbstwirksamkeitserwartungen von angehenden Lehrkräften

Im Modell professioneller Handlungskompetenz von Lehrkräften (Baumert und Kunter 2006) gelten Lehrerselbstwirksamkeitserwartungen als bedeutsame Komponente motivationaler Orientierungen. Die sozial-kognitive Theorie nach Bandura (1997) beschreibt Selbstwirksamkeitserwartungen als individuelle Überzeugungen, eigene Handlungen erfolgreich initiieren und aufrechterhalten zu können, um schwierige Situationen zu bewältigen. Lehrerselbstwirksamkeit bezieht sich dabei unter anderem auf die Überzeugung, unterrichtsbezogene Herausforderungen auch angesichts von Schwierigkeiten erfolgreich bewältigen zu können (Tschannen-Moran und Woolfolk Hoy 2001). Empirische Befunde zeigen, dass hohe Selbstwirksamkeitserwartungen es Lehrkräften ermöglichen, qualitätsvollen Unterricht durchzuführen (Schwarzer und Jerusalem 2002; Zee und Koomen 2016). Die Entstehung von Selbstwirksamkeitsüberzeugungen wird dabei als zyklischer Prozess verstanden: Zu den zentralen Quellen der Selbstwirksamkeit zählen erfolgreiches Handeln, stellvertretende Erfahrungen, verbale Persuasion oder affektive sowie physiologische Erregung – eigene (Lehr‑)Erfahrungen werden zunächst entlang dieses Kontinuums kognitiv bewertet und dieser Bewertungsprozess beeinflusst, wie neue (Lehr‑)Aufgaben in Relation zu den eigenen Lehrkompetenzen beurteilt werden. Aus diesem Beurteilungsprozess generiert sich das eigene Selbstwirksamkeitserleben, das sich wiederum auf die eigenen Zielsetzungen und die Anstrengung, die in die Erreichung der gesetzten Ziele investiert wird, auswirkt. Aus diesem Prozess folgende (Lehr‑)Erfahrungen wirken sich wiederum auf das Erleben wichtiger Quellen der Selbstwirksamkeit wie erfolgreiches Handeln oder affektive Erregung aus. Auf theoretischer Ebene wird angenommen, dass hohe Selbstwirksamkeitserwartungen dazu führen, dass Lehrkräfte sich herausfordernde Ziele für ihre Unterrichtsgestaltung setzen, sich daraufhin stärker mit neuen und innovativen Lehrmethoden auseinandersetzen und auch positiv auf die Bereitschaft wirken, neue Methoden und Unterrichtsstrategien in herausfordernden Unterrichtssituationen umzusetzen (vgl. Tschannen-Moran et al. 1998). Tschannen-Moran und Hoy (2001) unterscheiden dabei drei Facetten von Lehrerselbstwirksamkeit: *Selbstwirksamkeit für Instruktionsstrategien, Selbstwirksamkeit für Schülerinnen- und Schülerengagement und Selbstwirksamkeit für Klassenmanagement*.

Hinsichtlich der Quellen von Selbstwirksamkeit – Erfolgserfahrungen (*mastery experiences*), Beobachtung von erfolgreichen Verhaltensmodellen (*vicarious experiences*), Feedback (*verbal persuasion*) und erlebte emotionale Zustände (*affective arousal*) (vgl. Morris et al. 2017) spielt die kognitive Verarbeitung von eigenen oder bei anderen beobachteten (stellvertretenden) Erfolgs- und Misserfolgserlebnissen eine zentrale Rolle für die Entwicklung von Selbstwirksamkeit (Bandura 1997; Schwarzer und Jerusalem 2002). Auch Unterrichtshospitationen können als Lernen am Modell aufgefasst werden, wenn beispielsweise die hospitierende Person die Lehrkraft dabei beobachtet, wie neue Methoden im Unterricht eingesetzt und diese für den eigenen Unterricht in Betracht gezogen werden (Urton 2017). Darüber hinaus gelten konstruktives Feedback von Dozierenden und Mentoren bzw. Mentorinnen (im Sinne verbaler Persuasion, Clark und Newberry 2019) sowie das Ausmaß an physiologischer und emotionaler Aktivierung als Quelle für die Entwicklung von Selbstwirksamkeit im Lehramtsstudium (Bandura 1997; Pfitzner-Eden 2016). Forschungsarbeiten, die sich mit der Selbstwirksamkeit von Lehramtsstudierenden befassen, konnten dementsprechend auch einen positiven Zusammenhang zwischen der Auseinandersetzung mit fremden sowie eigenen Unterrichtsvideos und dem Kompetenzerleben Studierender feststellen (Gold et al. 2017). Im Hinblick auf die Veränderung der Selbstwirksamkeit bei Lehramtsstudierenden zeigen bisherige Studien vorwiegend einen Anstieg der Selbstwirksamkeit zu Studienbeginn (z. B. Lamote und Engels 2010) sowie während des Studiums vor allem im Verlauf von bzw. nach schulpraktischen Phasen (z. B. Garvis et al. 2012; Schüle et al. 2017a). Dennoch ist die Forschungslage bezüglich der Veränderungen der Selbstwirksamkeitserwartungen während des Lehramtsstudiums und darüber hinaus noch immer inkonsistent (Schüle et al. 2017a), da auch auf einen university shock verwiesen wird, den Studierenden zu Beginn des Studiums erleben können und der zu einem Absinken des Selbstwirksamkeitserlebens führen kann (Pfitzner-Eden 2016). Es wird davon ausgegangen, dass Selbstwirksamkeitserwartungen anfänglich leichter veränderbar sind und mit zunehmenden Erfolgserfahrungen immer stabiler werden, da Lehrkräfte und Lehramtsstudierende ihre Erfolgserfahrungen in einer spezifischen Situation auch auf andere Situationen übertragen können und annehmen, auch in diesen Situationen erfolgreich handeln zu können (Bandura 1977; Woolfolk Hoy und Spero 2005).

### Selbstwirksamkeitserwartungen während Praxisphasen in der Lehrkräftebildung

Praxisphasen können mit einem Anstieg von Selbstwirksamkeitserwartungen einhergehen (Klassen und Durksen 2014) – erklärende Faktoren dafür sind unterstützende Anleitungen von Mentorinnen und Mentoren (*verbal persuasion*) (Klassen und Durksen 2014; Moulding et al. 2014) sowie eigene Unterrichtserfahrungen (*mastery experiences*) (Cantrell et al. 2003). Auch Rollenspiele und Mikroteaching-Erfahrungen mit spezifischem Feedback besitzen einen Einfluss auf die Selbstwahrnehmung der Lehrkräftekompetenz (Tschannen-Moran et al. 1998). Zudem kann davon ausgegangen werden, dass stellvertretende Erfolgserfahrungen (*vicarious experiences*) in Unterrichtshospitationen bei erfolgreichen Lehrpersonen im positiven Zusammenhang mit der Selbstwirksamkeit angehender Lehrkräfte stehen (Bandura 1997; Palmer 2006; Tatar und Buldur 2013). Empirische Studien zeigen, dass Lehramtsstudierende Praxisphasen als Herausforderung wahrnehmen und diejenigen Studierenden, die positive Veränderungen erleben, berichten, dass gerade die erfolgreiche Bewältigung dieser herausfordernden Studienphase als stärkend für die eigene unterrichtsbezogene Selbstwirksamkeit empfunden wird (Klassen und Durksen 2014). Dieser Befund ist im Einklang mit der Annahme von Bandura (1997), dass leicht zu bewältigende Aufgaben weniger selbstwirksamkeitsrelevant sind als herausfordernde Aufgaben. Neben Forschungsarbeiten, die einen Anstieg der Selbstwirksamkeit von Lehramtsstudierenden nach Praxisphasen berichten (Eisfeld et al. 2020; Fives et al. 2007; Ronfeldt und Reininger 2012; Schüle et al. 2017a), wird in anderen Studien jedoch auch auf eine Stabilität der Selbstwirksamkeit von Studierenden bzw. auf eine negative Korrelation der Anzahl von Unterrichtserfahrungen im Rahmen von Praxisphasen verwiesen (Capa Aydin und Woolfolk Hoy 2005; Lin und Gorrell 2001 ). Mögliche Erklärungen dieser inkonsistenten Befunde werden in Unterschieden im Studiendesign bzw. der Erhebungsmethoden z. B. in Bezug auf den Umfang der Praktikumsdauer vermutet (Bach 2013). Einige Studien zeigten einen Anstieg der Lehrer-Selbstwirksamkeit nach einem Schulpraktikum, die jedoch im Verlauf des ersten Berufsjahres wieder absinkt (Woolfolk Hoy und Spero 2005).

Ein wichtiges Anliegen von Praxisphasen ist darüber hinaus, dass sich Lehramtsstudierende einen Habitus des forschenden Lernens – im Sinne des Modells des *reflective practitioner* nach Schön (1983) – aneignen. Diese Fähigkeit zur kritischen Reflexion kann günstig auf die Entwicklung der unterrichtsbezogenen Selbstwirksamkeit wirken (Black 2015). Allerdings ist wenig darüber bekannt, inwiefern Praxisphasen einen Einfluss auf die Selbstwirksamkeit von Lehramtsstudierenden in Bezug auf das Reflektieren verschiedener Unterrichtshandlungen besitzen. Weß et al. (2018) konnten zeigen, dass die Selbstwirksamkeit zur Reflexion bei Studierenden nach mindestens einem Praktikum signifikant höher war als bei Studierenden ohne Praktikum (*d* = 0,19). Somit kann angenommen werden, dass Praxisphasen das Kompetenzerleben im Bereich der Reflexion besonders anregen. Insgesamt sind die spezifischen Bedingungen, unter denen sich Selbstwirksamkeit im Verlauf der Lehrkräftebildung verändert, bisher jedoch unzureichend empirisch untersucht worden (Clark und Newberry 2019).

### Beanspruchungserleben von angehenden Lehrkräften

In der aktuellen Forschung wird zwischen Belastungen und Beanspruchungen unterschieden – während Belastungen als objektive, von außen auf Individuen einwirkende Prozesse und Faktoren gelten, werden Beanspruchungen als interindividuell variierende Auswirkungen objektiver Belastungen definiert (Rudow 1995). Eine psychische Beanspruchung resultiert aus der Einschätzung, dass externe Anforderungen bedrohlich sind oder die individuellen Bewältigungsressourcen übersteigen (Lazarus und Folkman 1984). Durch die wiederholte Konfrontation mit solchen Stressoren können Symptome von Burnout entstehen (Rudow 1995). Das Burnout-Syndrom wurde von Maslach et al. (1996) als ein dreidimensionales Phänomen aus emotionaler Erschöpfung, Depersonalisierung und Ineffektivität (verringerter persönlicher Leistungsfähigkeit) beschrieben. In anderen Publikationen wird der letztgenannte Faktor auch im Sinne positiv formulierter Überzeugungen einer professionellen Wirksamkeit (*professional efficacy*) erfasst (z. B. Schaufeli und Salanova 2007). *Emotionale Erschöpfung* bezeichnet eine gefühlsmäßige Überforderung. *Depersonalisierung* (Zynismus) wird durch eine distanzierte und durch Abwertung charakterisierte Einstellung gegenüber Personen des Arbeitsgeschehens beschrieben. *Professionelle Wirksamkeit* ist durch das Gefühl charakterisiert, Arbeitsanforderungen erfolgreich bewältigen zu können.^1^

Besonders im Lehrberuf begünstigen stressfördernde Arbeitsaufgaben und soziale Gegebenheiten ein hohes Belastungserleben (Rothland 2013). Bisherige Studien konnten zeigen, dass Lehramtsstudierende bereits während ihres Studiums psychisch belastet sind (Bauer 2019; Römer et al. 2012) – insbesondere durch Aspekte wie Prüfungsleistungen, zu viele Anforderungen in zu kurzer Zeit oder der Koordinierung von Nebenjobs und Studium (Kosinár und Leineweber 2010). Darüber hinaus konnte Bauer (2019) feststellen, dass die personalen Gesundheitsressourcen der Lehramtsstudierenden wie Selbstwirksamkeit oder Achtsamkeit als bessere Prädikatoren der psychischen Beanspruchung gelten als soziodemografische bzw. studiumsbezogene Charakteristika.

### Beanspruchung während Praxisphasen in der Lehrkräftebildung

Zahlreiche Studien untersuchen das Beanspruchungserleben während Praxisphasen im Lehramtsstudium – allerdings sind die Ergebnisse eher inkonsistent. Es wird davon ausgegangen, dass mangelnde Unterrichtserfahrungen und Fähigkeiten im Unterrichten dazu führen können, dass die vielfältigen Belastungen in Praxisphasen als besonders beanspruchend erlebt werden (Bauer 2019). In der Studie von Holtz (2014) berichten die Studierenden, dass das Beanspruchungserleben im Praxissemester höher ist als in anderen Phasen des Studiums. Studierende berichten aber auch, dass sie die überdurchschnittlichen Belastungen nicht als Bedrohung, sondern als wertvolle Herausforderung wahrnehmen (Bauer 2019). Auch Längsschnittstudien, die Studierende im Verlauf von Praxisphasen mehrfach zu ihrem Belastungserleben befragen, finden unterschiedliche Verläufe. Einige Studien verweisen auf einen Anstieg des Beanspruchungserlebens während des Praxissemesters bzw. im Vorbereitungsdienst (Pereira Kastens et al. 2020; Schüle et al. 2017b). Andere Studien deuten auf eine Abnahme des Beanspruchungserlebens im Verlauf des Praxissemesters hin (Krawiec et al. 2020; Römer et al. 2018). Eine mögliche Erklärung für solche inkonsistenten Befunde könnte in praxisbegleitenden Faktoren liegen – so wirken beispielsweise Handlungsspielräume oder soziale Unterstützung (Gusy et al. 2016), die Anzahl der Unterrichtsstunden oder die Unterstützung durch Mentorinnen und Mentoren (Kücholl et al. 2019) sowie ein guter Kontakt mit Schülerinnen und Schülern (Timoštšuk und Ugaste 2012) dem Beanspruchungserleben in Praxisphasen entgegen. Zu hohe universitäre Anforderungen sowie problematische Interaktionen mit betreuenden Lehrkräften bzw. Dozierenden wirken sich wiederum negativ auf das Beanspruchungserleben der Studierenden aus (Timoštšuk und Ugaste 2012).

### Online-Lehre in Schule und Hochschule

Studien, die sich mit den Auswirkungen des COVID-19-bedingten Fernunterrichts befassen, zeigen ein hohes Beanspruchungserleben seitens der Lernenden im schulischen Fernunterricht (Huber et al. 2020), insbesondere bei Schülerinnen und Schülern, deren Eltern einen niedrigen Bildungsabschluss oder einen Migrationshintergrund besitzen bzw. die auf engem Raum leben (Ravens-Sieberer et al. 2021). Besonders individuelle Unterstützung und Rückmeldung sind für die Motivation und den Lernfortschritt im schulischen Fernunterricht bedeutend und sind sogar über den Einfluss des sozioökonomischen Status hinaus relevant (Steinmayr et al. 2021). Bisherige Studien zur pandemiebedingten Online-Lehre bei Studierenden zeigen, dass besonders die genutzte Technik, die Organisation sowie die Qualität der Instruktion oder der Umgang mit Lehrenden als herausfordernd wahrgenommen werden (vgl. Schmölz et al. 2020). In deskriptiven Umfragen an deutschen Universitäten berichtet eine Vielzahl der befragten Studierenden, dass sich die Arbeitsbelastung während der Online-Lehre im Gegensatz zur regulären Lehre erhöht hat (Stefanica et al. 2021; Traus et al. 2020). Auch andere Untersuchungen der COVID-19-bedingten Online-Lehre deuten darauf hin, dass Studierende Schwierigkeiten mit der situativ angepassten Lehre aufweisen (Traus et al. 2020; Van Nguyen et al. 2020). Faktoren, wie z. B. die Strukturierung bzw. die zeitliche Organisation der Arbeit zu Hause, Misserfolgsangst oder ressourcenintensivere Lernaktivitäten werden als erklärende Einflussfaktoren für ein erhöhtes Beanspruchungserleben der Studierenden während der Online-Lehre angesehen (Hahn et al. 2021; Stefanica et al. 2021). Neben den neuen Herausforderungen in der Online-Lehre fehlt gleichzeitig ein schulpraktischer Anteil, der von Lehramtsstudierenden teilweise als Herausforderung, aber teilweise auch als besonders beanspruchend wahrgenommen wird. Bislang existieren kaum empirische Studien, die sich vor diesem Hintergrund der Frage nach der Veränderung des Beanspruchungserlebens während der COVID-19-bedingten Online-Lehre in der Lehrkräftebildung widmen.

### Die vorliegende Studie

Empirische Studien konnten zeigen, dass der COVID-19-bedingte schulische Fernunterricht eine hohe Belastung der Schülerinnen und Schüler zur Folge hat (z. B. Huber et al. 2020). Hinsichtlich der Kompetenzentwicklung der Lernenden während der Pandemie zeigt sich eine unklare Befundlage – einige Studien belegten eine ungünstigere Kompetenzentwicklung durch den Distanzunterricht während der Pandemie (Lernende der Sekundarstufe: Dorn et al. 2020; Lernende der Primarstufe: Tomasik et al. 2020), gleichzeitig konnte dies nicht studien- und altersgruppenübergreifend nachgewiesen werden (keine langsameren Kompetenzzuwächse bei Lernenden der Sekundarstufe: Tomasik et al. 2020; keine Kohortenunterschiede zwischen Präsenz- und Distanzunterricht in Primar- und Sekundarstufe: Depping et al. 2021). Auch in der Lehrkräftebildung ist zu erwarten, dass das Online-Format bedeutend für die Selbstwirksamkeitserwartungen und das Beanspruchungserleben der Studierenden im Semesterverlauf ist. Aufgrund der fehlenden Praxiselemente in der Online-Lehre haben Lehramtsstudierende nicht die Möglichkeit, Erfolgserfahrungen zu sammeln und konstruktives Feedback zum eigenen Unterricht zu erhalten – allerdings stellen diese Faktoren potenzielle Quellen der Selbstwirksamkeit dar (Clark und Newberry 2019). Sie erleben zudem keinen erhöhten emotionalen Arousal während der realen Unterrichtspraxis, der auch als Quelle der Selbstwirksamkeit gilt (Morris et al. 2017). Die Beobachtung von erfolgreichen Verhaltensmodellen, die ebenfalls günstig für die Entwicklung von Selbstwirksamkeitserwartungen sein kann, ist jedoch auch in der Online-Lehre möglich. Andererseits müssen sich Lehramtsstudierende im Online-Semester nicht den vielfältigen Herausforderungen, wie unklaren Rollenzuweisungen in der Schulpraxis stellen, die potenziell als emotional erschöpfend erlebt werden (Krawiec et al. 2020). Aktuell beschäftigen sich nur wenige empirische Arbeiten mit den Effekten der Online-Lehre in der Lehrkräftebildung (z. B. Traus et al. 2020). Vor diesem Hintergrund untersucht die vorliegende Studie die differentielle Veränderung von Selbstwirksamkeitserwartungen und Beanspruchungserleben bei Lehramtsstudierenden im Verlauf von Präsenzsemester und Online-Semester.

Folgende Forschungsfragen und Hypothesen werden in der Studie untersucht:

#### Fragestellungen 1a und 1b:

Wie verändern sich Selbstwirksamkeitserwartungen von Lehramtsstudierenden im Semesterverlauf? Welche Unterschiede in der Veränderung der Selbstwirksamkeit zeigen sich zwischen den Lehramtsstudierenden in den Präsenzsemestern und im Online-Semester?

#### Hypothese 1a:

Insgesamt wird von einem Anstieg der Selbstwirksamkeitserwartungen von Lehramtsstudierenden im Semesterverlauf ausgegangen.

#### Hypothese 1b:

Es wird angenommen, dass der Anstieg der Selbstwirksamkeitserwartungen aufgrund der eigenen sowie stellvertretenden Praxiserfahrungen und der verbalen Überzeugungen sowie der emotionalen Zustände im Präsenzsemester mit Praxisanteil stärker ausgeprägt ist als im Online-Semester.

#### Fragestellungen 2:

Wie verändert sich das Beanspruchungserleben von Lehramtsstudierenden im Semesterverlauf in der Präsenzsemestergruppe und in der Online-Semestergruppe?

## Methode

### Stichprobenbeschreibung

Die vorliegende Studie basiert auf längsschnittlichen Fragebogendaten von insgesamt *N* = 240 Lehramtsstudierenden einer deutschen Universität (54,6 % weiblich; 88,8 % in Deutschland geboren), die jeweils zu Beginn und zum Ende des Semesters an einem Online-Survey teilnahmen. Ein Teil der Studierenden besuchte Onlineseminare im Sommersemester 2020 (Gruppe „Online-Semester“: *n* = 127; 52,76 % weiblich; *M*_Alter_ = 23,75; *SD* = 4,12). Ein weiterer Teil der Lehramtsstudierenden wurde im Sommersemester 2019 bzw. im Wintersemester 2019/2020 befragt (Gruppe „Präsenzsemester“: *n* = 113 Lehramtsstudierende; 56,64 % weiblich; *M*_Alter_ = 23,80; *SD* = 3,24)^2^. Die in den Präsenzsemestern durchgeführten Seminare mit schulpraktischem Anteil beinhalteten Unterrichtsversuche, in denen die Lehramtsstudierenden zunächst bei der betreuenden Lehrkraft während einer Unterrichtsstunde (90 min) hospitieren, darauffolgend eine Unterrichtsstunde (90 min) im Seminar konzipieren und vorstellen, um im Anschluss Feedback von Studierenden und Dozierenden bezüglich der Planung und Umsetzung der Entwürfe zu erhalten. Anschließend wird der eigene Unterrichtsentwurf in einer der kooperierenden Schulen in einer Unterrichtsstunde (90 min) praktisch umgesetzt. Schließlich werden die Unterrichtserfahrungen im Seminar systematisch über mehrere Sitzungen reflektiert. Der durch die COVID-19-bedingte Ausfall des praktischen Anteils des Seminars im Online-Semester wurde durch praxisnahe Übungen ersetzt. Sowohl in der Online-Semestergruppe als auch in der Präsenzsemestergruppe wurden im Rahmen bildungswissenschaftlicher Seminare die Themen *Unterrichtsqualität* und *Motivierender Unterricht* behandelt, die in der Studienordnung am Ende des Bachelorstudiums verortet sind. Die Studierenden aus beiden Gruppen befanden sich zum Zeitpunkt der Befragung überwiegend im 4. bis 5. Fachsemester (Online-Semester: *M* = 4,28; *SD* = 1,88; Präsenzsemester: *M* = 5,20; *SD* = 2,35). Die vier häufigsten Erstfächer der befragten Studierenden im Online-Semester waren Sport (19,69 %), Deutsch (17,32 %), Englisch (10,24 %) und Geschichte (10,24 %). Die drei am häufigsten studierten Erstfächer der Studierenden in den Präsenzsemestern waren Deutsch (20,35 %), Englisch (12,39 %) und Sport (10,62 %).

### Messinstrumente

#### Selbstwirksamkeitserwartungen angehender Lehrkräfte

Die Selbstwirksamkeitserwartungen der Studierenden in beiden Gruppen wurden anhand von drei Subskalen (*Instruktionsstrategien, Klassenmanagement, Engagement für Schülerinnen und Schüler*) mit validierten deutschsprachigen Messinstrumenten (Pfitzner-Eden et al. 2014) der Originalskalen von Tschannen und Woolfolk Hoy (2001) erhoben (siehe Tab. 1). Jede der drei Subskalen beinhaltet vier Items mit sechsstufigem Antwortformat von 1 (trifft überhaupt nicht zu) bis 6 (trifft voll und ganz zu). Die internen Konsistenzen waren insgesamt akzeptabel bis gut (vgl. Tab. 1). Aufgrund der Reflexionsphase der Unterrichtserfahrungen der Studierenden und der Annahme der resultierenden unterstützenden Rollenfindung durch Mentorinnen bzw. Mentoren (Brombach 2019) sowie hinsichtlich der intensiven Betreuung der Studierenden insbesondere in der Reflexion der Lernumgebung als Maßnahmen zur Selbstwirksamkeitsstabilisierung nach Bandura (1997) wurde zusätzlich die Skala *Reflexionsbezogene Selbstwirksamkeitserwartung* von Fraij (2018) verwendet. Die Skala umfasst fünf Items mit einem Antwortformat 1 (trifft überhaupt nicht zu) bis 6 (trifft voll und ganz zu). Die Reliabilität der Skala ist zu allen Zeitpunkten akzeptabel bis gut (siehe Tab. 1).

**Table 1:** 

Skala	Konstrukt	Beispielitem	Interne Konsistenz	
			Online-Semester	Präsenzsemester
Selbstwirksamkeitserwartungen [SWE] (Pfitzner-Eden et al. 2014)	SWE bzgl. Instruktionsstrategien	„Ich bin davon überzeugt, eine alternative Erklärung oder ein anderes Beispiel finden zu können, wenn die Lernenden etwas nicht verstehen.“	α_T1_ = 0,78; α_T2_ = 0,85	α_T1_ = 0,69; α_T2_ = 0,82
	SWE bzgl. Engagement für Schülerinnen und Schüler	„Ich bin davon überzeugt, die Lernenden, die wenig Interesse am Unterricht haben, motivieren zu können.“	α_T1_ = 0,80; α_T2_ = 0,82	α_T1_ = 0,70; α_T2_ = 0,82
	SWE bzgl. Klassenmanagement	„Ich bin davon überzeugt, störendes Verhalten im Unterricht kontrollieren zu können.“	α_T1_ = 0,86; α_T2_ = 0,87	α_T1_ = 0,78; α_T2_ = 0,85
Reflexionsbezogene Selbstwirksamkeitserwartungen (Fraij 2018)	SWE bzgl. Reflexion	„Ich kann mir vorhergegangene problematische Situationen noch einmal vorstellen, um zu überlegen, wie ich hätte besser handeln können.“	α_T1_ = 0,82; α_T2_ = 0,86	α_T1_ = 0,76; α_T2_ = 0,81

#### Beanspruchungserleben angehendender Lehrkräfte

Zur Erfassung der Beanspruchung bzw. des Burnouts der Lehramtsstudierenden während des Semesters wurden die Subskalen des Maslach-Burnout-Inventorys in der Studierendenversion (MBI-SS) von Schaufeli et al. (2002) erhoben (siehe Tab. 2). Das MBI-SS umfasst die drei Subskalen *Emotionale Erschöpfung, Depersonalisierung* und *professionelle Wirksamkeit*. Das Antwortformat der drei Subskalen reicht von 1 (stimmt nicht) bis 4 (stimmt genau). Die internen Konsistenzen sind insgesamt als akzeptabel bis sehr gut einzuschätzen (vgl. Tab. 2).

**Table 2:** 

Skala	Konstrukte	Beispielitem	Interne Konsistenz	
			Online-Semester	Präsenzsemester
Beanspruchung/Burnout (Schaufeli et al. 2002)	Emotionale Erschöpfung	„Durch mein Studium bin ich gefühlsmäßig am Ende.“	α_T1_ = 0,85 α_T2_ = 0,89	α_T1_ = 0,87 α_T2_ = 0,85
	Depersonalisierung (Zynismus)	„Ich zweifele inzwischen stärker an der Nützlichkeit meines Studiums.“	α_T1, T2_ = 0,85	α_T1_ = 0,82 α_T2_ = 0,90
	Professionelle Wirksamkeit	„Ich habe während meines Studiums viele interessante Dinge gelernt.“	α_T1_ = 0,67 α_T2_ = 0,79	α_T1_ = 0,75 α_T2_ = 0,73

### Statistische Analysen

Im Rahmen von Varianzanalysen mit Messwiederholung wurden Unterschiede im mittleren Niveau der Selbstwirksamkeitserwartungen und im Beanspruchungserleben zu allen Zeitpunkten sowie Unterschiede in der Veränderung dieser Merkmale im Semesterverlauf in beiden Gruppen (Online- versus Präsenzsemester) untersucht. Als Innersubjektfaktor wurde der Faktor Zeit (T_1_: Beginn des Semesters; T_2_: Ende des Semesters) und als Zwischensubjektfaktor wurde die Gruppenzugehörigkeit (Online- vs. Präsenzsemester) berücksichtigt.

In die Studie einbezogen wurde die Gesamtstichprobe der Studierenden, die entweder zum ersten oder zum zweiten Messzeitunkt an der Studie teilgenommen hatten. Zum ersten Messzeitpunkt (Beginn des Semesters) nahmen von den insgesamt *N* = 240 in die Analyse einbezogenen Lehramtsstudierenden 90,8 % (*n* = 218 Studierende) an der Befragung teil. Zum zweiten Zeitpunkt (Ende des Semesters) nahmen 81,6 % (*n* = 196) der Gesamtstichprobe an der Studie teil. In den Hauptanalysen wurden alle Studierenden mit fehlenden Werten auf den einbezogenen Variablen ausgeschlossen („listenweiser Fallausschluss“). Eine Analyse fehlender Werte zu beiden Messzeitpunkten mit den einbezogenen Messinstrumenten (vier Selbstwirksamkeitsskalen und drei Burnout-Skalen zu je zwei Messzeitpunkten) zeigte, dass kein systematisches Fehlen der Werte vorlag (siehe Appendix, Tab. 7).

## Ergebnisse

### Deskriptive Daten

Die in Tab. 3 dargestellten Mittelwerte der Selbstwirksamkeitserwartungen deuten bereits darauf hin, dass in der Gruppe Präsenzsemester ein tendenzieller Anstieg der Selbstwirksamkeitserwartungen stattfand, während in der Gruppe Online-Semester die Selbstwirksamkeitserwartungen teilweise sanken. Die Ergebnisse weisen zudem auf einen ähnlich starken Anstieg des Beanspruchungserlebens bei den Studierenden im Präsenzsemester und den Studierenden im Online-Semester hin. Die Korrelationen zwischen den Subskalen der Konstrukte Selbstwirksamkeitserwartungen und Beanspruchung bzw. Burnout sind in Tab. 4 dargestellt. Die Ergebnisse der Korrelationen deuten darauf hin, dass in der Präsenzsemestergruppe eine geringere zeitliche Rangstabilität des Selbstwirksamkeitserlebens als in der Online-Semestergruppe vorlag – vor allem traf dies auf die Selbstwirksamkeit für Instruktionsstrategien zu (Präsenz: *r* = 0,170, *p* = 0,150; Online: *r* = 0,651, *p* < 0,001) sowie auf die Selbstwirksamkeit für Klassenmanagement (Präsenz: *r* = 0,180, *p* = 0,128; Online: *r* = 0,596, *p* < 0,001). Die drei Burnout-Skalen *Emotionale Erschöpfung* (BE_EE_), *Depersonalisierung* (BE_DP_) und *Professionelle Wirksamkeit* (BE_PW_) wiesen in beiden Gruppen hohe Korrelationen über die Zeit hinweg auf (Präsenzsemestergruppe: BE_EE_:* r* = 0,613, *p* < 0,001; BE_DP_:* r* = 0,574, *p* < 0,001; BE_PW_:* r* = 0,680, *p* < 0,001; Online-Semestergruppe: BE_EE_: *r* = 0,747, *p* < 0,001; BE_DP_: *r* = 0,778, *p* < 0,001; BE_PW_:* r* = 0,637, *p* < 0,001). Diese korrelativen Befunde deuten darauf hin, dass in der Online-Semestergruppe die Rangstabilität in allen Konstrukten relativ hoch war und Personen, die zu Semesterbeginn ein höheres Selbstwirksamkeitserleben und geringeres Burnout-Erleben berichteten, auch am Semesterende günstigere Ausprägungen erzielten. Von den drei Burnout-Skalen war es die Skala Professionelle Wirksamkeit, die am stärksten mit den Selbstwirksamkeitsfacetten korrelierte bzw. Depersonalisierung war geringer und überwiegend nicht signifikant (siehe Tab. 4).

**Table 3:** 

	Online-Semester				Präsenzsemester			
	T_1_		T_2_		T_1_		T_2_	
	*M*	*SD*	*M*	*SD*	*M*	*SD*	*M*	*SD*
*Selbstwirksamkeitserwartungen*								
Selbstwirksamkeit für Instruktionsstrategien	4,76	0,62	4,71	0,70	4,75	0,55	4,90	0,57
Selbstwirksamkeit für Klassenmanagement	4,59	0,70	4,62	0,68	4,45	0,58	4,77	0,65
Selbstwirksamkeit für Engagement für SuS ^a^	4,86	0,65	4,73	0,64	4,72	0,57	4,91	0,57
Reflexionsbezogene Selbstwirksamkeit	5,16	0,58	5,05	0,66	5,06	0,56	5,15	0,54
*Beanspruchung (Burnout)*								
Emotionale Erschöpfung	1,96	0,67	2,05	0,72	1,94	0,66	1,98	0,64
Depersonalisierung (Zynismus)	1,76	0,75	1,77	0,75	1,68	0,68	1,80	0,79
Professionelle Wirksamkeit	3,10	0,39	3,07	0,47	3,11	0,47	3,13	0,45

^a^ Schülerinnen und Schüler

**Table 4:** 

	BE_EET1_	BE_DPT1_	BE_PWT1_	SW_IST1_	SW_KMT1_	SW_EST1_	SW_RT1_	BE_EET2_	BE_DPT2_	BE_PWT2_	SW_IST2_	SW_KMT2_	SW_EST2_	SW_RT2_
BE_EET1_	1	0,374^*^	−0,273^*^	−0,081	−0,044	0,016	0,037	0,613^*^	0,219	−0,166	−0,151	−0,307^*^	−0,218	−0,273^*^
BE_DPT1_	0,468^*^	1	−0,422^*^	−0,179	−0,122	−0,163	−0,011	0,148	0,574^*^	−0,300^*^	−0,235^*^	−0,309^*^	−0,218	−0,208
BE_PWT1_	−0,338^*^	−0,477^*^	1	0,199^*^	0,126	0,239^*^	0,285^*^	−0,230	−0,439^*^	0,680^*^	0,193	0,218	0,243^*^	0,370^*^
SW_IST1_	−0,090	−0,066	0,286^*^	1	0,405^*^	0,550^*^	0,279^*^	0,072	−0,006	0,064	0,170	0,171	0,328^*^	0,124
SW_KMT1_	−0,192^*^	−0,155	0,256^*^	0,430^*^	1	0,491^*^	0,109	−0,110	0,030	−0,050	0,230^*^	0,180	0,122	0,054
SW_EST1_	−0,144	−0,154	0,343^*^	0,551^*^	0,552^*^	1	0,418^*^	−0,085	−0,076	0,000	0,239^*^	0,198	0,307^*^	0,128
SW_RT1_	−0,165	−0,107	0,299^*^	0,355^*^	0,438^*^	0,501^*^	1	0,021	−0,018	0,234^*^	0,258^*^	0,086	0,251^*^	0,437^*^
BE_EET2_	0,747^*^	0,402^*^	−0,309^*^	−0,143	−0,320^*^	−0,224^*^	−0,181	1	0,442^*^	−0,367^*^	−0,074	−0,173	−0,160	−0,257^*^
BE_DPT2_	0,413^*^	0,778^*^	−0,436^*^	−0,083	−0,158	−0,160	−0,170	0,447^*^	1	−0,458^*^	−0,196	−0,274^*^	−0,225^*^	−0,171
BE_PWT2_	−0,250^*^	−0,334^*^	0,637^*^	0,152	0,142	0,236^*^	0,186	−0,277^*^	−0,365^*^	1	0,335^*^	0,301^*^	0,407^*^	0,462^*^
SW_IST2_	−0,141	−0,231^*^	0,307^*^	0,651^*^	0,350^*^	0,452^*^	0,345^*^	−0,182	−0,146	0,252^*^	1	0,643^*^	0,769^*^	0,575^*^
SW_KMT2_	−0,126	−0,155	0,175	0,389^*^	0,596^*^	0,345^*^	0,283^*^	−0,200^*^	−0,106	0,172	0,565^*^	1	0,633^*^	0,491^*^
SW_EST2_	−0,130	−0,174	0,337^*^	0,524^*^	0,349^*^	0,582^*^	0,399^*^	−0,105	−0,046	0,254^*^	0,642^*^	0,472^*^	1	0,638^*^
SW_RT2_	−0,044	−0,148	0,302^*^	0,522^*^	0,335^*^	0,414^*^	0,509^*^	−0,121	−0,141	0,294^*^	0,659^*^	0,481^*^	0,647^*^	1

Anmerkungen: Unter der Diagonalen: COVID-19-bedingtes Online-Semester/Über der Diagonalen: Präsenzsemester mit Praxisanteil. Alle mit * markierten Korrelationen sind mindestens auf dem Niveau *p* < 0,05 signifikant. *BE*_*EE*_ Beanspruchung: Emotionale Erschöpfung, *BE*_*DP*_ Beanspruchung: Depersonalisierung, *BE*_*PW*_ Beanspruchung: Professionelle Wirksamkeit, *SW*_*IS*_ Selbstwirksamkeit für Instruktionsstrategien, *SW*_*KM*_ Selbstwirksamkeit für Klassenmanagement, *SW*_*ES*_ Selbstwirksamkeit für SchülerInnen-Engagement, *SW*_*R*_ Reflexionsbezogene Selbstwirksamkeit, *T1* zu Semesterbeginn, *T2* zu Semesterende

### Varianzanalysen mit Messwiederholung: Selbstwirksamkeitserwartungen

In einer 2 * 2‑faktoriellen Varianzanalyse mit Messwiederholung wurden die einzelnen Selbstwirksamkeitsskalen als abhängige Variablen durch den Innersubjektfaktor Zeit (T_1_: Beginn des Semesters; T_2_: Ende des Semesters) und den Zwischensubjektfaktor Gruppenzugehörigkeit (Online- versus Präsenzsemester) prädiziert. Die Ergebnisse der Analysen sind in Tab. 5 verdeutlicht.

**Table 5:** 

Selbstwirksamkeit	Zeit (A)	Gruppe (B)	A × B	
SW_IS_	0,67	3,64	2,48	*F*
	0,415	0,058	0,117	*p*
	0,004	0,020	0,014	η^2^
SW_KM_	17,37	0,07	5,28	*F*
	**0,000**	0,785	**0,023**	*p*
	0,089	0,000	0,029	η^2^
SW_ES_	1,57	0,12	14,24	*F*
	0,213	0,656	**0,000**	*p*
	0,009	0,001	0,074	η^2^
SW_R_	0,50	0,11	5,20	*F*
	0,823	0,918	**0,024**	*p*
	0,000	0,000	0,028	η^2^

^a^ zusätzliche Analysen von Interaktionseffekten zwischen der Zeit und den Kovariaten Geschlecht, Alter, Abiturnote bzw. Geburtsland zeigen überwiegend keine signifikanten Effekte

Für die Subskala *Selbstwirksamkeit für Instruktionsstrategien* (SW_IS_) zeigten sich in der Varianzanalyse keine Haupteffekte des Messwiederholungsfaktors Zeit als Innersubjektfaktor, *F*(1, 179) = 0,67, *p* = 0,415, η^2^ = 0,004 nach Greenhouse-Geisser-Korrektur sowie des Zwischensubjektfaktors Gruppe, *F*(1, 179) = 3,64, *p* = 0,058, η^2^ = 0,020. Weiterhin war die Interaktion Zeit * Gruppe, *F*(1, 179) = 2,48, *p* = 0,117, η^2^ = 0,014 nicht signifikant. Die Fehlervarianzen waren in beiden Gruppen zu beiden Messzeitpunkten gemäß Levene-Test für alle Variablen der Subskala (SW_IS_) homogen (*p* > 0,05).

**Figure 1:**
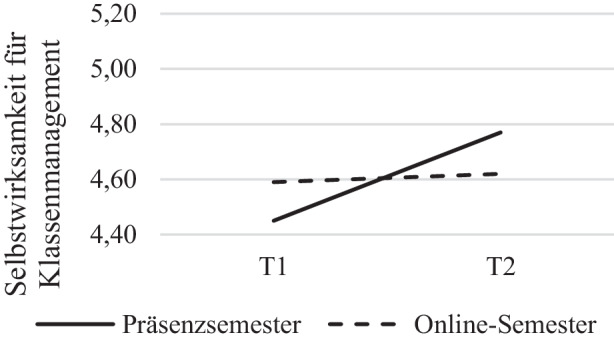

Für die Subskala* Selbstwirksamkeit für Klassenmanagement* (SW_KM_) zeigten sich höchstsignifikante Effekte des Innersubjektfaktors Zeit, *F*(1, 178) = 17,37, *p* < 0,001, η^2^ = 0,089, nach Greenhouse-Geisser-Korrektur jedoch keine signifikanten Ergebnisse des Zwischensubjektfaktors Gruppe, *F*(1, 178) = 0,07, *p* = 0,785, η^2^ = 0,000. Für die Interaktion Zeit * Gruppe, *F*(1, 178) = 5,28, *p* = 0,023, η^2^ = 0,029 zeigten sich hingegen signifikante Ergebnisse (siehe Abb. 1). Dabei stieg die Selbstwirksamkeit für Klassenmanagement bei den Studierenden des Präsenzsemesters von Semesterbeginn zum Semesterende stärker an (*M*_Präsenz ∆T1, T2_ = 0,32) als bei den Studierenden im Online-Semester, bei denen kein signifikanter Zuwachs zu verzeichnen war (*M*_Online ∆T1, T2_ = 0,03; vgl. Tab. 3). Zusätzliche Varianzanalysen, bei denen der Haupteffekt der Zeit auf die Selbstwirksamkeit getrennt für beide Gruppen untersucht wurde, verdeutlichten einen signifikanten Effekt des Innersubjektfaktors Zeit in der Präsenzsemestergruppe auf die Selbstwirksamkeit für Klassenmanagement, nicht jedoch im Online-Semester (Online-Semester: *F*(1, 109) = 1,01, *p* = 0,317, η^2^ = 0,009; Präsenzsemester: *F*(1, 85) = 19,79, *p* < 0,001, η^2^ = 0,189). Die Fehlervarianzen waren in beiden Gruppen zu beiden Messzeitpunkten gemäß Levene-Test für alle Variablen der Subskala (SW_KM_) homogen (*p* > 0,05).

**Figure 2:**
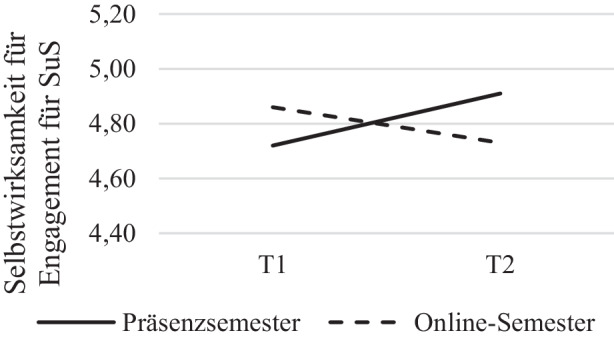

Für die Subskala *Selbstwirksamkeit im Engagement für Schülerinnen und Schüler* (SW_ES_) als abhängige Variable zeigten sich keine signifikanten Effekte des Innersubjektfaktors Zeit, *F*(1, 179) = 1,57, *p* = 0,213, η^2^ = 0,009, nach Greenhouse-Geisser-Korrektur und keine signifikanten Ergebnisse des Zwischensubjektfaktors Gruppe, *F*(1, 179) = 0,12, *p* = 0,656, η^2^ = 0,001. Für die Interaktion Zeit * Gruppe, *F*(1, 179) = 14,24, *p* < 0,001, η^2^ = 0,074 ergaben sich, nach Greenhouse-Geisser-Korrektur jedoch höchstsignifikante Ergebnisse (siehe Abb. 2). Die Selbstwirksamkeit im Engagement für Schülerinnen und Schüler bei den Studierenden stieg im Präsenzsemester von Semesterbeginn zu Semesterende an (*M*_Präsenz ∆T1, T2_ = 0,19) und sank im Verlauf des Online-Semesters (*M*_Online ∆T1, T2_ = −0,13; vgl. Tab. 1). Zusätzliche Varianzanalysen, bei denen der Haupteffekt der Zeit auf die Selbstwirksamkeit getrennt für beide Gruppen untersucht wurde, zeigten, dass der Innersubjektfaktor Zeit in beiden Gruppen signifikant auf die Selbstwirksamkeit für Engagement für Schülerinnen und Schüler wirkt (Online-Semester: *F*(1, 107) = 4,46, *p* = 0,037, η^2^ = 0,400; Präsenzsemester: *F*(1, 85) = 12,53, *p* < 0,001, η^2^ = 0,128). Die Fehlervarianzen waren in beiden Gruppen zu beiden Messzeitpunkten gemäß Levene-Test für alle Variablen der Subskala (SW_ES_) homogen (*p* > 0,05).

**Figure 3:**
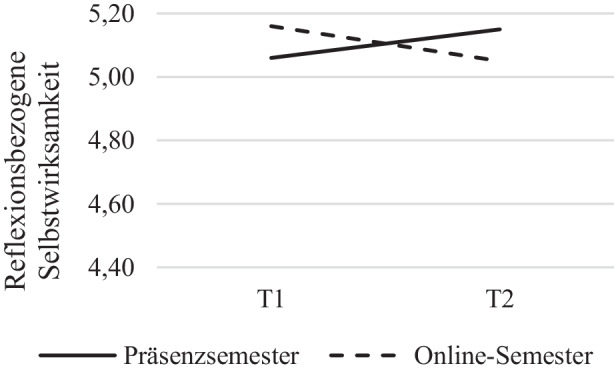

Die für die Skala *Reflexionsbezogene Selbstwirksamkeit* (SW_R_) als abhängige Variable durchgeführte 2 * 2‑faktoriellen Varianzanalyse mit Messwiederholung weist keine signifikanten Ergebnisse für den Faktor Zeit auf *F*(1, 179) = 0,050, *p* = 0,823, η^2^ = 0,000, nach Greenhouse-Geisser-Korrektur. Der Zwischensubjektfaktor Gruppe zeigte ebenfalls keine signifikanten Ergebnisse, *F*(1, 179) = 0,11, *p* = 0,918, η^2^ = 0,000. Für die Interaktion Zeit * Gruppe, *F*(1, 179) = 5,20, *p* = 0,024, η^2^ = 0,028 ergaben sich jedoch signifikante Ergebnisse (siehe Abb. 3). Dabei stieg die Reflexionsbezogene Selbstwirksamkeit bei den Studierenden des Präsenzsemesters von Semesterbeginn zum Semesterende an (*M*_Präsenz ∆T1, T2_ = 0,09) wohingegen bei den Studierenden im Online-Semester ein Abwärtstrend zu verzeichnen war (*M*_Online ∆T1, T2_ = −0,11; vgl. Tab. 3). Zusätzliche Varianzanalysen, bei denen der Haupteffekt der Zeit auf die Selbstwirksamkeit getrennt für beide Gruppen untersucht wurde, zeigten, dass die Zeit in der Präsenzsemestergruppe – nicht jedoch im Online-Semester – signifikant auf die Reflexionsbezogene Selbstwirksamkeit wirkte (Online-Semester: *F*(1, 107) = 2,56, *p* = 0,113, η^2^ = 0,023; Präsenzsemester: *F*(1, 85) = 5,00, *p* = 0,028, η^2^ = 0,056). Die Fehlervarianzen waren in beiden Gruppen zu beiden Messzeitpunkten gemäß Levene-Test für alle Variablen der Subskala (SW_R_) homogen (*p* > 0,05).

### Varianzanalysen mit Messwiederholung: Beanspruchungserleben

In weiteren 2 * 2‑faktoriellen Varianzanalysen mit Messwiederholung wurden die Subskalen des Beanspruchungserleben durch den Innersubjektfaktor Zeit (T_1_: Beginn des Semesters; T_2_: Ende des Semesters) und den Zwischensubjektfaktor Gruppenzugehörigkeit (Online- versus Präsenzsemester) vorhergesagt (siehe Tab. 6).

**Table 6:** 

Beanspruchung	Zeit (A)	Gruppe (B)	A × B	
BE_EE_	5,13	0,78	0,21	*F*
	**0,025**	0,378	0,647	*p*
	0,028	0,004	0,001	η^2^
BE_DP_	2,39	0,03	1,77	*F*
	0,124	0,858	0,185	*p*
	0,013	0,000	0,010	η^2^
BE_PW_	1,20	0,74	0,04	*F*
	0,275	0,390	0,855	*p*
	0,007	0,004	0,000	η^2^

^a^ zusätzliche Analysen von Interaktionseffekten zwischen den hier genannten Hauptkriterien und den Kovariaten Geschlecht, Alter, Abiturnote bzw. Geburtsland zeigen überwiegend keine signifikanten Effekte

Für die Subskala *Emotionale Erschöpfung* (BE_EE_) zeigten sich signifikante Effekte des Innersubjektfaktors Zeit, *F*(1, 179) = 5,13, *p* = 0,025, η^2^ = 0,028, nach Greenhouse-Geisser-Korrektur. Die Emotionale Erschöpfung der Studierenden in beiden Gruppen stieg signifikant über das Semester hinweg an (vgl. Tab. 1). Der Zwischensubjektfaktor Gruppe erwies sich nicht als signifikant, *F*(1, 179) = 0,78, *p* = 0,378, η^2^ = 0,004. Für die Interaktion Zeit * Gruppe ergab sich keine signifikante Interaktion, *F*(1, 179) = 0,21, *p* = 0,647, η^2^ = 0,001. Die Fehlervarianzen waren in beiden Gruppen zu beiden Messzeitpunkten gemäß Levene-Test für alle Variablen der Subskala (BE_EE_) homogen (*p* > 0,05).

Für die Subskala *Depersonalisierung* (BE_DP_) zeigten sich keine signifikanten Effekte der Zeit, *F*(1, 179) = 2,39, *p* = 0,124, η^2^ = 0,013 und keine signifikanten Effekte des Zwischensubjektfaktors Gruppe, *F*(1, 179) = 0,03, *p* = 0,858, η^2^ = 0,000. Für die Interaktion Zeit * Gruppe,* F*(1, 179) = 1,77, *p* = 0,185, η^2^ = 0,010, zeigten sich nach Greenhouse-Geisser-Korrektur ebenfalls keine signifikanten Ergebnisse. Die Fehlervarianzen waren in beiden Gruppen zu beiden Messzeitpunkten gemäß Levene-Test für alle Variablen der Subskala (BE_DP_) homogen (*p* > 0,05).

Auch für die Subskala *professionelle Wirksamkeit* (BE_PW_) zeigte sich kein signifikanter Effekt der Zeit, *F*(1, 179) = 1,20, *p* = 0,275, η^2^ = 0,007, nach Greenhouse-Geisser-Korrektur und des Zwischensubjektfaktors Gruppe, *F*(1, 178) = 0,74, *p* = 0,390, η^2^ = 0,004. Darüber hinaus war die Interaktion Zeit * Gruppe nicht signifikant, *F*(1, 179) = 0,04, *p* = 0,855, η^2^ = 0,000. Die Fehlervarianzen waren in beiden Gruppen zu beiden Messzeitpunkten gemäß Levene-Test für alle Variablen der Subskala (BE_PW_) homogen (*p* > 0,05).

## Diskussion

Nur wenige Studien befassen sich bislang mit den Auswirkungen der Online-Lehre auf sozio-emotionale und motivationale Merkmale von Lehramtsstudierenden (z. B. Osterberg et al. 2020). Insbesondere Selbstwirksamkeitserwartungen werden als bedeutsamer Einflussfaktor auf den Umgang mit Belastungen beschrieben (Klusmann et al. 2012). Forschung zu den Effekten verschiedener Lehr-Lernsettings auf die Entwicklung solcher Kompetenzüberzeugungen sowie des Beanspruchungserlebens ist daher von hoher Relevanz für die aktuelle Lehrkräftebildung. Vor diesem Hintergrund hatte die vorliegende Studie zum Ziel, die Veränderung von Selbstwirksamkeitserwartungen und Beanspruchungserleben im Semesterverlauf unter Differenzierung von Präsenzlehre und digitaler Lehre in Zeiten der COVID-19-Pandemie zu untersuchen.

### Selbstwirksamkeitserwartungen im Online- und im Präsenzsemester

Unsere Ergebnisse zeigen, dass sich drei der vier untersuchten Facetten der *Selbstwirksamkeitserwartungen* von Lehramtsstudierenden – konkret Selbstwirksamkeit für Klassenmanagement, im Engagement für Schülerinnen und Schüler und in Bezug auf Reflexion des Unterrichtsgeschehens – im COVID-19-bedingten Online-Semester und im Präsenzsemester differentiell entwickeln. Bei den Studierenden im Online-Semester blieben die Selbstwirksamkeit im Klassenmanagement und die Reflexionsbezogene Selbstwirksamkeit im Verlauf des Semesters stabil, wohingegen diese bei den Studierenden in den Präsenzsemestern im Semesterverlauf anstieg. Darüber hinaus sank die Selbstwirksamkeit im Engagement für Schülerinnen und Schüler im Online-Semester, während sie im Verlauf des Präsenzsemesters anstieg. Mögliche Erklärungen für die günstigere Entwicklung der drei Selbstwirksamkeits-Facetten im Präsenzsemester könnten erfolgreiche Praxiserfahrungen (*mastery experiences*) sowie die systematische Unterstützung und das konstruktive Feedback von erfahrenen Lehrkräften im Sinne verbaler Bestärkung durch andere Personen (*verbal persuasion*) in der Lehre mit praktischem Anteil sein (Bandura 1977; Morris et al. 2017). Sowohl die Auseinandersetzung mit Unterrichtserfahrungen als auch unterstützendes Feedback gelten als prädiktiv für die Lehrerselbstwirksamkeit wie Studien bei angehenden Lehrkräften zeigen konnten (Richter et al. 2011; Ronfeldt und Reininger 2012). Studierende im Präsenzsemester konnten außerdem auch ihre physiologische Aktivierung durch das Unterrichten wahrnehmen, die als bedeutend als Quelle von Selbstwirksamkeit angesehen wird (Snyder und Fisk 2016), was den Studierenden im Online-Semester aufgrund der fehlenden Praxisanteile nicht möglich war. Die differentielle Veränderung unterschiedlicher Selbstwirksamkeitsfacetten im Online-Semester könnte durch die Art der praxisbezogenen Anteile im Online-Semester bedingt gewesen sein. Diese praktischen Übungen bezogen sich insbesondere auch auf die Arbeit mit Unterrichtsvideografien – dabei sind Merkmale der Klassenführung für Studierende eventuell besser beobachtbar und beurteilbar als beispielsweise Motivierungsstrategien der Lehrkraft (Kunter und Baumert 2006). Stellvertretende Erfolgserfahrungen (*vicarious experiences*) als Quelle der Selbstwirksamkeit waren daher insbesondere im Bereich der Motivierung von Lernenden eventuell nur wenig verfügbar, während ein erfolgreicher Umgang mit Störungen auf Unterrichtsvideographien besser für die Lehramtsstudierenden beobachtbar war. Allerdings sind diese Erklärungsansätze für die differentielle Veränderung der einzelnen Selbstwirksamkeitsfacetten nur hypothetisch und sollten in weiterführenden Untersuchungen differenzierter in den Blick genommen werden.

Eine mögliche Erklärung für die nicht-signifikanten Effekte auf die Selbstwirksamkeit für Instruktionsstrategien könnte einerseits darin bestehen, dass erfolgreiche Instruktion häufig nur langfristig wahrnehmbar ist, während sich ein gezieltes Eingreifen bei Störungen oder eine erfolgreiche motivierende Unterrichtsführung, die das Schülerinnen- und Schülerengagement befördert, in ihren Konsequenzen eher unmittelbar wahrnehmen lassen (Luttenberger et al. 2019; Ophardt und Thiel 2017). Zudem könnte das systematische Reflektieren eigener kurzer Unterrichtserfahrungen im Präsenzsemester zum Anstieg der Selbstwirksamkeit im Reflektieren beigetragen haben (Stürmer et al. 2013).

### Beanspruchungserleben im Online- und im Präsenzsemester

Unsere Befunde verweisen auf ähnliche Veränderungen der Facetten des *Beanspruchungserlebens* im Präsenz- und Online-Semester. Gleichzeitig zeigte sich ein Anstieg der Emotionalen Erschöpfung in beiden Gruppen, während Depersonalisierung und professionelle Wirksamkeit in beiden Gruppen stabil blieben. Sowohl in Praxisphasen als auch während der pandemiebedingten Online-Lehre gibt es spezifische universitäre Anforderungen, die von den Studierenden als belastend erlebt werden (Bach 2015; Hahn et al. 2021). In Praxisphasen erleben Studierende vor allem schwieriges Verhalten von Schülerinnen und Schülern, die Organisation und Betreuung des Praktikums sowie ambivalente Rollendefinitionen als emotional erschöpfend (Krawiec et al. 2020). Allerdings ist die Befundlage hinsichtlich der Veränderungen emotionaler Erschöpfung bei Lehramtsstudierenden in Praxisphasen inkonsistent, da Studien teilweise auf einen Anstieg der Erschöpfung verweisen (Schüle et al. 2017b), während andere Studien Rückgänge der emotionalen Erschöpfung aufzeigen (Fives et al. 2007). In der pandemiebedingten Online-Lehre werden Faktoren wie die zeitliche Organisation und Strukturierung der Arbeit zu Hause sowie Misserfolgsängste und ressourcenintensivere Lernaktivitäten von den Studierenden als emotional erschöpfend empfunden (Hahn et al. 2021). In unserer Untersuchung waren die Studierenden daher sowohl im Semester mit Praxisanteil als auch im Online-Semester mit spezifischen und für sie neuen universitären Anforderungen konfrontiert, die emotional erschöpfend gewirkt haben können. Um der emotionalen Erschöpfung von Lehramtsstudierenden im Semesterverlauf entgegenzuwirken, wären sowohl in der Online-Lehre als auch während Praxisphasen unterstützende Anleitungen durch Mentoren und Mentorinnen bzw. Dozierende (Klassen und Durksen 2014) bzw. die Förderung der Unterstützung zwischen den Studierenden möglich (Römer et al. 2018). Für die Belastungs-Facetten Depersonalisierung und professionelle Wirksamkeit zeigen unsere Ergebnisse keine Zunahme im Semesterverlauf. Dieses Befundmuster ist im Einklang mit früheren Studien zum Referendariat, die darauf verweisen, dass insbesondere die emotionale Erschöpfung in längeren Praxisphasen des Lehramtsstudiums ansteigt (Klusmann et al. 2012), während die professionelle Wirksamkeit stabil bleibt (Zimmermann et al. 2016). Paradox an unseren Befunden mag erscheinen, dass im Präsenzsemester mit Praxisanteil die emotionale Erschöpfung zwar im Semesterverlauf anstieg, aber auch die Selbstwirksamkeit – in drei von vier Facetten – anstieg. Klassen und Durksen (2014) konnten diesen Verlauf bei einigen Studierenden ihrer Stichprobe ebenfalls beobachten und führen diese Befunde auf hohe Belastungen zurück, die aber durch günstige Bewältigungsstrategien in Form sozialer Unterstützung durch Familie und Freunde bewältigt werden konnten. Da auch Unterstützungsangebote durch Kommilitonen und Kommilitoninnen sowie durch Mentoren und Mentorinnen als Prädiktoren für die emotionale Erschöpfung fungieren können (Römer et al. 2018), wäre eine mögliche Implikation unserer Befunde, dass eine verstärkte systematische Förderung der Kooperation zwischen den Studierenden sowohl in Präsenz- als auch in Online-Formaten sinnvoll wäre. Allerdings müsste die Wirksamkeit solcher Maßnahmen gesondert in zukünftigen Studien untersucht werden.

### Limitationen

Unsere Untersuchung weist einige Limitationen auf. Zunächst liegen uns keine Daten aus einem Online-Semester vor, das nicht zusätzlich durch COVID-19 bedingt war. Daher können wir Belastungen, die durch das Online-Format der Lehrveranstaltungen entstehen, nicht klar von Belastungen durch die COVID-19-Pandemie trennen. Da in der bisherigen Lehrkräftebildung reine Online-Veranstaltungsformate sehr selten waren, sollten weiterführende Untersuchungen prüfen, wie sich Selbstwirksamkeit und Belastungserleben von Lehramtsstudierenden in digitalen Veranstaltungen auch jenseits der COVID-19-Pandemie entwickeln. Unsere Untersuchung lässt offen, welche Relevanz verschiedene Wirkmechanismen – fehlende Erfolgserfahrungen und fehlende positive Rückmeldungen (Bandura 1997), höhere Arbeitsbelastung (Traus et al. 2020; Zentrum für Qualitätsentwicklung in Lehre und Studium 2020) und möglicherweise auch der geringere Grad an sozialer Eingebundenheit – für die differentielle Veränderung von Selbstwirksamkeit und Beanspruchung im Online-Seminar verglichen mit dem Präsenzseminar haben. Darüber hinaus konnte nicht eindeutig festgestellt werden, welche Rolle die Praxisanteile im Präsenzsemester in Bezug auf die Veränderung der Selbstwirksamkeit und der Beanspruchung der Studierenden gespielt haben. Die differentiellen Veränderungen sowohl der Selbstwirksamkeit als auch des Beanspruchungserlebens sind daher nur eingeschränkt auf andere Veranstaltungsformate mit zeitlich umfangreicheren Praxisanteilen, auf andere Stichproben (Zeitpunkt der Praxisanteile im Studienverlauf bzw. Fachsemester der Studierenden) bzw. auf andere Universitäten oder Länder übertragbar. Eine weitere Limitation der Studie liegt im eingesetzten Analyseverfahren, da keine tatsächlichen Veränderungen, sondern lediglich Gruppenunterschiede untersucht werden konnten. Zukünftig sollten die Ergebnisse der vorliegenden Studie anhand größerer Samples validiert werden, wobei auch elaboriertere Verfahren latenter Differenzenmodelle zum Einsatz kommen sollten, die Messfehler berücksichtigen und Differenzen so akkurater abbilden können.

### Implikationen und Ausblick

Angesichts der COVID-19-bedingten Umstellung der Lehrkräftebildung auf Online-Formate in den vergangenen Monaten sind empirische Ergebnisse zur Entwicklung der professionellen Kompetenz von Lehramtsstudierenden im Rahmen dieser Veranstaltungsformate von großer Relevanz. Unsere Untersuchung liefert erste Hinweise, dass sich insbesondere Selbstwirksamkeitserwartungen im Klassenmanagement, im Engagement für Schülerinnen und Schüler und in den Reflexionsbezogenen Selbstwirksamkeitserwartungen in Veranstaltungen im Präsenzformat mit Praxisanteilen im Semesterverlauf deutlicher entwickeln als in Online-Seminaren ohne Praxisanteile, in dem sogar ein Absinken zu verzeichnen ist. Dennoch bedarf es weiterer empirischer Untersuchungen, um die Veränderung von Selbstwirksamkeitserwartungen von Lehramtsstudierenden in verschiedenen Veranstaltungsformaten nachzuzeichnen und die Rolle von Einflussfaktoren, wie Zeitpunkt des Praktikums im Studium, Dauer, Aufgaben, Reflexion und Betreuung an der Schule sowie der Hochschule, herauszuarbeiten (Ding 2020).

## Anhang

**Table 7:**

Skala	**χ**^2^ für Missing Value^a^	*p*
*Selbstwirksamkeitserwartungen*		
Selbstwirksamkeit für Instruktionsstrategien	1,19	0,553
Selbstwirksamkeit für Klassenmanagement	0,89	0,640
Selbstwirksamkeit im Schülerinnen- und Schülerengagement	1,63	0,443
Reflexionsbezogene Selbstwirksamkeitserwartungen	2,26	0,323
*Burnout*		
Emotionale Erschöpfung	1,13	0,568
Depersonalisierung (Zynismus)	2,26	0,324
Professionelle Wirksamkeit	1,12	0,571

^**a**^ mit Fachsemester

## Footnotes

1 Die Items werden in manchen Arbeiten rekodiert und als *reduzierte Leistungsfähigkeit* benannt (Schaufeli und Salanova 2007), in anderen Arbeiten werden die Items nicht rekodiert und im Sinne einer *professional efficacy* erfasst (Bresó et al. 2007). In der vorliegenden Studie nutzen wir das positiv formulierte Konstrukt.

2 2 Die in der Studie einbezogenen Konstrukte aus den zusammengefassten Kohorten (Sommersemester 2019 und dem Wintersemester 19/20) in der Präsenzsemestergruppe unterschieden sich nicht signifikant zwischen den Kohorten zu T1.
